# Robust Learning Control for Shipborne Manipulator With Fuzzy Neural Network

**DOI:** 10.3389/fnbot.2019.00011

**Published:** 2019-04-04

**Authors:** Zhiqiang Xu, Wanli Li, Yanran Wang

**Affiliations:** School of Mechanical Engineering, Tongji University, Shanghai, China

**Keywords:** shipborne manipulator, real-time adaptive control, conventional PD controller, fuzzy neural network, sliding mode control, experiment verification

## Abstract

The shipborne manipulator plays an important role in autonomous collaboration between marine vehicles. In real applications, a conventional proportional-derivative (PD) controller is not suitable for the shipborne manipulator to conduct safe and accurate operations under ocean conditions, due to its bad tracing performance. This paper presents a real-time and adaptive control approach for the shipborne manipulator to achieve position control. This novel control approach consists of a conventional PD controller and fuzzy neural network (FNN), which work in parallel to realize PD+FNN control. Qualitative and quantitative tests of simulations and real experiments show that the proposed PD+FNN controller achieves better performance in comparison with the conventional PD controller, in the presence of uncertainty and disturbance. The presented PD+FNN eliminates the requirements for precise tuning of the conventional PD controller under different ocean conditions, as well as an accurate dynamics model of the shipborne manipulator. In addition, it effectively implements a sliding mode control (SMC) theory-based learning algorithm, for fast and robust control, which does not require matrix inversions or partial derivatives. Furthermore, simulation and experimental results show that the angle compensation deviation of the shipborne manipulator can be improved in the range of ±1°.

## 1. Introduction

The shipborne manipulator has become the most important tool in achieving autonomous cargo reloading between marine vehicles. With the use of the shipborne manipulator, onboard physical labor can be greatly reduced. However, unpredictable ship motion has a great impact on the maneuverability of the manipulator in real applications due to the complexity of the marine environment. If the sea state reaches Level-4, i.e., the height of a sea wave is larger than 1.52 m and wind speed exceeds 10.8 m/s, arm movement of the manipulator is extremely limited because of the influence of sling inertia and non-linear ship pose variation. In this case, the operational capacity of the manipulator is reduced by more than 50% or the manipulator is even temporarily suspended.

**Figure 1:**
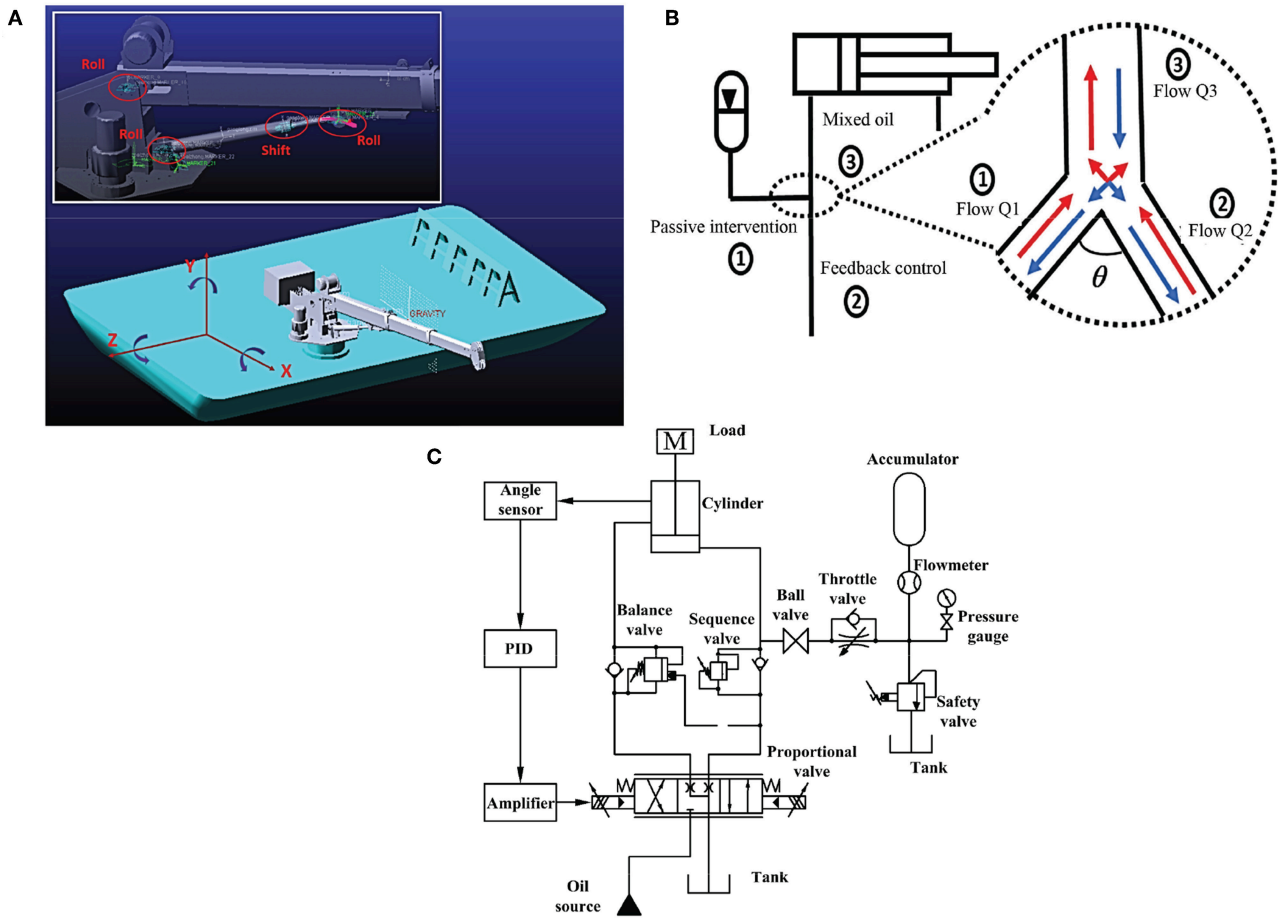
Cylinder control strategy with the intervention of an accumulator, **(A)** is the joints of manipulator, **(B)** is the flow direction indication, **(C)** is the hydraulic system diagram with control signals.

For the shipborne manipulator, changing boom inclination is realized through the expansion and contraction of its amplitude cylinder. The energy-saving and vibration-damping function of its accumulator is of great significance for improving manipulator control. In literature, the cylinder-accumulator in a manipulator has been studied in different types of engineering applications (Xiao et al., 2014; Shen et al., 2015; Zhao et al., 2017; Xia et al., 2018). Specifically, the accumulator is a key component in the design of hydraulic hybrid structures, which ensures acceptable shock absorption performance and system energy consumption. Its different designs are used in other construction machines such as a rock drill (Yang et al., 2017), Scraper (Junke and Zhen, 2017), and Fast Forging Press (Zhang et al., 2016). However, there are few investigations related to the influence of the accumulator on the valve control system. According to the valve-controlled cylinder-accumulator model, the accumulator is used as an energy-saving and oil damping source in parallel with the rodless cavity of the amplitude cylinder. Figure 1 shows the details of connecting the accumulator with the cylinder.

In literature, the conventional PD controller is often used to control different types of manipulators (Cervantes and Alvarez-Ramirez, 2001; Alvarez-Ramirez et al., 2003; Su et al., 2010). However, it is not suitable for controlling the hydraulic system discussed in this work, due to its high-order non-linearity, time-varying and hysteresis characteristics. It cannot control the hydraulic system in time and is vulnerable to environmental interference. Additionally, a significant steady-state error still exists, even if plenty of time is used to tune the appropriate values for the conventional PD controller. In order to control the manipulator, calculating torque is the simplest control approach, but this approach relies on the accurate mechanical model of the system. To overcome this issue, the model-free approach has gained respectable attention since it does not require the precise model of the system and is more robust in response to uncertainty and disturbance.

The fuzzy logic controller (FLC) is widely applied to handle uncertainty and disturbance in many systems (Hasanien and Matar, 2015; Dabbaghjamanesh et al., 2016; Vaidyanathan and Azar, 2016), especially in different kinds of robots (Fu et al., 2013; Tai et al., 2016; Sarabakha et al., 2018). However, the FLC also requires a lot of time in order to tune the proper parameters to achieve a satisfied control performance. Recently, the FLC has been combined with an artificial neural network (ANN), i.e., fuzzy neural network (FNN), to overcome the aforementioned weakness of the FLC. In literature, the FNN has been successfully applied in identification and non-linear system controlling (Lin et al., 2015; Tang et al., 2017; He and Dong, 2018). At the same time, the sliding mode control (SMC) theory-based algorithm has been presented as a faster learning approach for tuning the FNN parameters, due to its faster convergence speed and higher robustness to uncertainty and disturbance (Lin et al., 2014). Moreover, the FNN, trained with the SMC theory-based algorithm, has been successfully used in controlling a spherical rolling robot (Kayacan et al., 2013), a robotic arm (Wai and Muthusamy, 2013), and a gyroscope (Yan et al., 2017).

In this work, an FNN is proposed to work in parallel with a conventional PD controller to achieve a PD+FNN controller. It not only overcomes the original defects of conventional PD control but also significantly enhances self-learning abilities and adaptabilities. In order to validate the proposed control strategy, simulation and experimental tests have been implemented. The qualitative and quantitative results show that the presented strategy is feasible and practical. In addition, it outperforms the conventional PD controller. The main contributions of this work are listed below:

- Designing a novel control strategy for real-time control of shipborne manipulator. The presented control strategy consists of a conventional PD controller and FNN, which combines a fuzzy logic controller and artificial neural network.
- Developing online adaptation laws to eliminate the requirement for precise tuning of the controller in the shipborne manipulator.
- Qualitative and quantitative tests in the simulation and real experiments have been conducted to evaluate the control performance of the presented PD+FNN control strategy.

The organization of this paper is as follows: In section 2, the dynamic model of the shipborne manipulator is introduced. In section 3, the PD+FNN control strategy is described. In section 4, different simulation tests are conducted in order to verify the proposed control strategy. In section 5, the real experimental tests on the swaying platform are performed to validate the proposed controller. Finally, conclusions are drawn in section 6.

## 2. Dynamic Model

**Figure 2:**
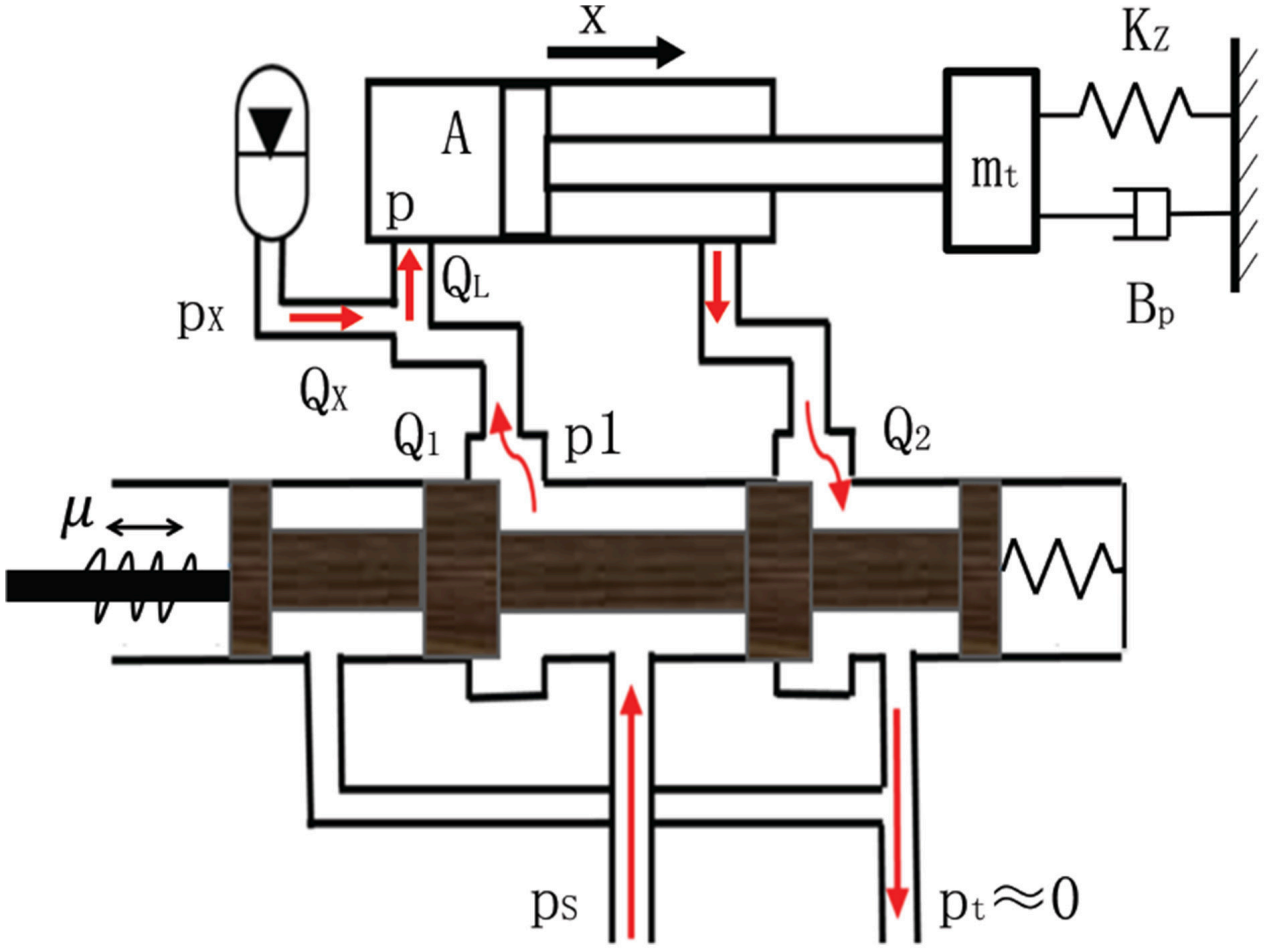
Connection schematic diagram of valve and accumulator.

Table 1 shows the simulation parameters. Figure 2 shows the flow direction of the oil. The accumulator is linked with the rodless cavity of the cylinder. When the cylinder extends, the accumulator and the valve supply oil to the cylinder simultaneously, making the cylinder stretch out faster. The coupling dynamics model of the parallel accumulator of the valve-controlled cylinder system is established based on the flow continuity equation and the dynamic equation.

**Table 1:** Simulation parameters.

**Parameter**	**Description**	**Value**	**Unites**
**name**			
*A*	action area of rodless cavity	6.36 × 10^−3^	*m*^*2*^
*m*_*t*_	mass of the piston	7.0	*kg*
*B*_*P*_	viscosity damping coefficient	0.2	*N*·*s/m*
*k*_*z*_	spring stiffness	8.0 × 10^2^	*N/m*
*C*_*i*_	leakage coefficient	5.1 × 10^−7^	*m*^*5*^/*N*·*s*
*k*_*q*_	flow gain coefficient	0.868	*m*^*2*^/*s*
*k*_*v*_	proportional coefficient of core	10^−3^	

### 2.1. Cylinder Dynamic Equation

The cylinder dynamic equation can be obtained by ignoring the cylinder cavity pressure, which is defined as:

(1) $$\begin{array}{l} pA=m_{t}ẍ+B_{P}ẋ+k_{z}x\;, \end{array}$$

where *p* denotes the working pressure, *N*/*m*^2^; *A* denotes the action area of rodless cavity, *m*^2^; *m*_*t*_ denotes the mass of the piston, *kg*; *x* denotes the displacement of pistol, *m*; *B*_*P*_ denotes the viscosity damping coefficient, *N* · *s*/*m*; *k*_*z*_ denotes the spring stiffness, *N*/*m*.

### 2.2. Cylinder Flow Equation

The cylinder flow continuity equation is:

(2) $$\begin{array}{l} Q_{L}=Aẋ+C_{i}p\;, \end{array}$$

where *C*_*i*_ denotes the internal leakage coefficient, *m*^5^/*N* · *s*; *Q*_*L*_ denotes the total flow into rodless cavity of cylinder, *m*^3^/*s*, which is defined as:

(3) $$\begin{array}{l} Q_{L}=Q_{1}+Q_{X}\;, \end{array}$$

where *Q*_1_ denotes the oil flow from valve, *m*^3^/*s*; *Q*_*X*_ denotes the oil flow from the accumulator, *m*^3^/*s*. Accumulator energy release process can be regarded as interference according to Gaussian distribution, i.e.,:

(4) $$\begin{array}{l} Q_{X}=N\left(\mu_{v},\sigma_{v}^{2}\right)\;, \end{array}$$

where μ_*v*_ and σ_*v*_ are the mean value and the standard deviation of the accumulator output flow, respectively.

### 2.3. Valve Flow Equation

The spool flow is a function of the working pressure and the displacement of the spool. The spool can be viewed as a zero-open four-way spool valve. The valve flow equation is defined as follows:

(5) $$\begin{array}{l} Q_{1}=C_{d}wx_{v}\sqrt{\frac{2}{\rho }\left(p_{S}-p_{1}\right)}=k_{q}x_{v}\;, \end{array}$$

where *C*_*d*_ denotes the flow coefficient of valve; *w* denotes the valve area gradient, *m*^2^/*m*; *x*_*v*_ denotes the displacement of valve core, *m*; *x*_*v*_ = *k*_*v*_·μ, *k*_*v*_ denotes the spool scale factor; μ denotes current signal; ρ denotes the oil density, kg/m^3^; ps denotes the system pressure, *N*/*m*^2^; *p*_1_ denotes the pressure of the rodless cavity, *N*/*m*^2^; *k*_*q*_ denotes the flow gain coefficient, *m*^2^/*s*, which is defined as:

(6) $$\begin{array}{l} k_{q}=C_{d}w\sqrt{\frac{2}{\rho }\left(p_{S}-p_{1}\right)}\;, \end{array}$$

Assume that the state of the system is *x*_1_ = *x*, *x*_2_ = ẋ, the dynamic model of the valve-controlled cylinder system can be defined as follows:

(7) $$\begin{array}{l} \{\begin{array}{l} {\dot{x}}_{1}=x_{2}\\ {\dot{x}}_{2}=\theta_{1}x_{1}+\theta_{2}x_{2}+g\mu +d\\ x=x_{1} \end{array}, \end{array}$$

where

(8) $$\begin{array}{l} \{\begin{array}{l} \theta_{1}=-\frac{k_{z}}{m_{t}}\\ \theta_{2}=-\left(\frac{A^{2}+C_{i}B_{P}}{m_{t}C_{i}}\right)\\ g=\frac{k_{q}k_{v}A}{m_{t}C_{i}}\\ d=\frac{N\left(\mu_{v},\sigma_{v}^{2}\right)A}{m_{t}C_{i}} \end{array}. \end{array}$$

## 3. PD+FNN Control Strategy

### 3.1. Overview of Control Strategy

**Figure 3:**
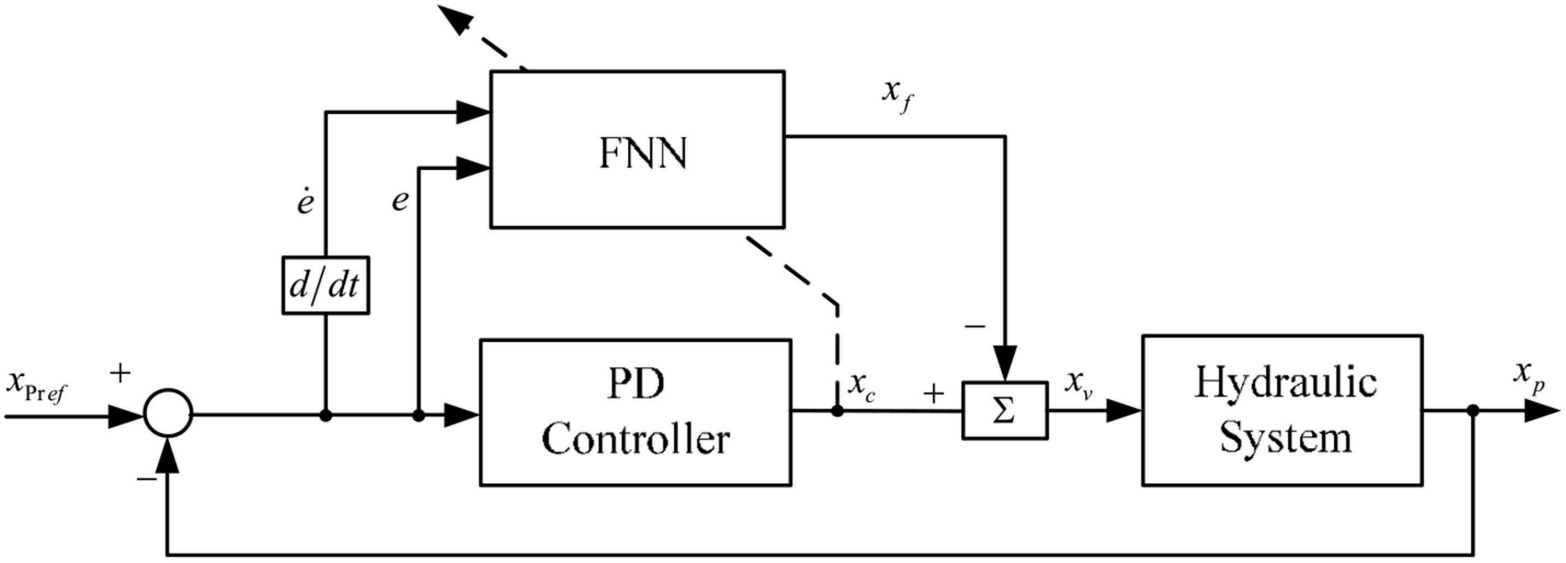
Presented PD+FNN control strategy for shipborne manipulator.

Figure 3 shows the presented PD+FNN control strategy, in which the conventional PD controller works in parallel with the fuzzy-neuro controller, as the FNN block shows in Figure 3. The PD controller is utilized to not only trace the target value by system error, i.e., *e* = *x*_*pref*_ − *x*_*p*_, but also to provide learning errors to train the FNN online. The FNN is supposed to improve the control accuracy and offset the effects of system interference.

### 3.2. Fuzzy Neural Network Construction

The proposed FNN consists of two input signals, i.e., *x*_1_ = *e* and *x*_2_ = *ė*, and one output signal *x*_*f*_. Takagi-Sugeno-Kang (TSK) fuzzy model (Lin et al., 2015; Precup et al., 2015) is used in which the antecedent part is the fuzzy set and the consequent part consists of only crisp numbers. The *r*th rule of a zero-order TSK model with two input variables *x*_1_ and *x*_2_ can be defined as follows:

(9) $$\begin{array}{l} \text{IF }x_{1}\text{ is }M_{1i}\text{ and }x_{2}\text{ is }M_{2j}\;,\text{ THEN }f_{ij}=d_{ij}\;, \end{array}$$

where *f*_*ij*_ is the time-varying parameter of the consequent part. *d*_*ij*_ is the coefficient of the output function for the *r*th rule, and *M*_1*i*_ and *M*_2*j*_ are fuzzy sets. Therefore, the inputs can be represented as μ_1*i*_ and μ_2*j*_, respectively. The firing strength of the *r*th rule is computed as the *T*-norm (multiplication) of the member functions (MFs) in the antecedent part (Imanberdiyev and Kayacan, 2018):

(10) $$\begin{array}{l} W_{ij}=\mu_{1i}\left(x_{1}\right)\mu_{2j}\left(x_{2}\right)\;, \end{array}$$

The output signal of the system can be derived using the normalized values of the firing strength ${\tilde{W}}_{ij}$ with the following form (Biglarbegian et al., 2010):

(11) $$\begin{array}{l} u_{f}=\overset{I}{\underset{i=1}{\sum }}\overset{J}{\underset{j=1}{\sum }}f_{ij}{\tilde{W}}_{ij}\;, \end{array}$$

where *J* and *I* represent the number of MFs for *x*_2_ and *x*_1_, respectively. ${\tilde{W}}_{ij}$ is expressed as follows:

(12) $$\begin{array}{l} {\tilde{W}}_{ij}=\frac{W_{ij}}{\sum_{i=1}^{I}\sum_{j=1}^{J}W_{ij}}\;. \end{array}$$

Overall control input *u* to the system is defined as follows:

(13) $$\begin{array}{l} x_{v}=x_{c}-x_{f}\;, \end{array}$$

where *x*_*c*_ and *x*_*f*_ are the control signals produced by the PD controller and the FNN controller, respectively.

### 3.3. Triangular Fuzzy MFs

In the FNN, the fuzzy MFs play the important role of overcoming environmental interference. These MFs have already shown promising results for control (Khanesar et al., 2015) and identification (Khanesar et al., 2011) purposes. In this work, typical triangular fuzzy MFs are chosen in order to achieve a faster and robust control performance. The MF is defined as follows:

(14) $$\begin{array}{l} \mu (x)=\{\begin{array}{ll} \left(1-\left|\frac{x-c}{d}\right|\right) & \text{if}\;c-d<x<c+d\\ 0 & \text{otherwise} \end{array}, \end{array}$$

where *x* is the input, *d* and *c* are the width and the center of the MF. The stability proof can be found in Kayacan and Khanesar (2015) according to sliding mode control theory.

### 3.4. Sliding Mode Control Theory-Based Training Approach

In this paper, SMC based parameter update rules are proposed to guarantee the stability of the system and provide favorable robustness. By utilizing the principles of the SMC theory, the zero dynamics of the learning error coordinate *x*_*c*_(*t*) can be described as a time-varying sliding surface *S*_*c*_ in the following form:

(15) $$\begin{array}{l} S_{c}\left(x_{f},x_{v}\right)=x_{c}\left(t\right)=x_{f}\left(t\right)+x_{v}\left(t\right)=0\;. \end{array}$$

If this condition is satisfied, the FNN structure is trained to become the non-linear regulator which assists the parallel controller (in our case PD controller), and the desired performance of the system can be obtained. Therefore, the sliding surface for the non-linear system under control is given by

(16) $$\begin{array}{l} S_{p}\left(e,ė\right)=ė+\chi e\;, \end{array}$$

with χ > 0 being a positive parameter which defines the desired trajectory of the error signal.

The time-varying parameter of the consequent part *ḟ_ij_* is updated based on the following adaptation law (Kayacan and Khanesar, 2015):

(17) $$\begin{array}{l} {ḟ}_{ij}=-\frac{{\tilde{W}}_{ij}}{\prod^{T}\prod }\alpha \text{sign}\left(x_{c}\right), \end{array}$$

where

(18) $$\begin{array}{l} \prod =\left(\overset{I}{\underset{i=1}{\sum }}\overset{J}{\underset{j=1}{\sum }}{\tilde{W}}_{ij}\right)\;, \end{array}$$

the learning rate α > 0 is updated based on the following equation:

(19) $$\begin{array}{l} \overset{•}{\alpha }=|x_{c}|, \end{array}$$

The adaptation law for the premise part is given as follows (Kayacan and Khanesar, 2015):

(20) $$\begin{array}{l} {ċ}_{1i}=-\gamma_{1}|d_{1i}|\left(1-T_{1i}\right)\text{sgn}\left(x_{1}-c_{1i}\right)\times H\left(x_{1},c_{1}-d_{1},c_{1}+d_{1}\right), \end{array}$$

(21) $$\begin{array}{l} {ḋ}_{1i}=-\gamma_{1}\frac{\left(1-T_{1i}\right)|d_{1i}|}{\left|x_{1}-c_{1i}\right|}\text{sgn}\left(d_{1i}\right)\times H\left(x_{1},c_{1}-d_{1},c_{1}+d_{1}\right), \end{array}$$

(22) $$\begin{array}{l} {ċ}_{2i}=-\gamma_{1}|d_{2j}|\left(1-T_{2j}\right)\text{sgn}\left(x_{2}-c_{2i}\right)\times H\left(x_{2},c_{2}-d_{2},c_{2}+d_{2}\right), \end{array}$$

(23) $$\begin{array}{l} {ḋ}_{2i}=-\gamma_{1}\frac{\left(1-T_{2j}\right)|d_{2j}|}{\left|x_{1}-c_{2j}\right|}\text{sgn}\left(d_{2j}\right)\times H\left(x_{2},c_{2}-d_{2},c_{2}+d_{2}\right), \end{array}$$

where

(24) $$\begin{array}{l} H(x,c,d)=\{\begin{array}{ll} x & \text{if}\;c-d<x<c+d\\ 0 & \text{otherwise} \end{array}, \end{array}$$

(25) $$\begin{array}{l} \{\begin{array}{l} T_{1,i}=\left|\frac{x_{1}-c_{i}}{d_{i}}\right|\\ T_{2,i}=\left|\frac{x_{2}-c_{i}}{d_{i}}\right| \end{array}. \end{array}$$

For the γ_1_, it needs to be selected as positive (Kayacan and Khanesar, 2015).

## 4. Simulation and Results Analysis

### 4.1. Simulation Parameter

The control gains for the PD controller are chosen as follows: *k*_*p*_ = 10, *k*_*d*_ = 5.

### 4.2. Simulation Results

**Figure 4:**
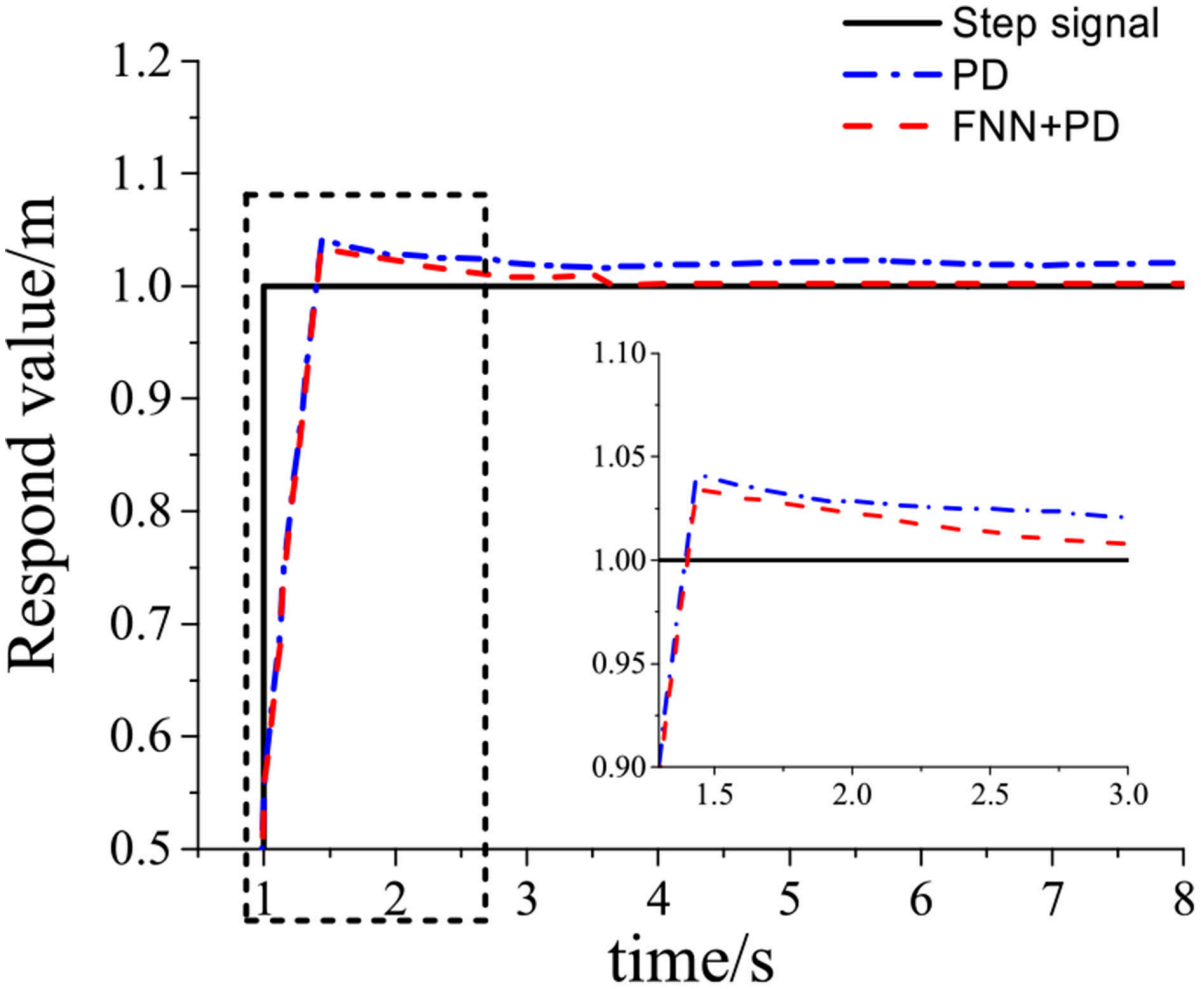
Tracking response of step signal.

**Figure 5:**
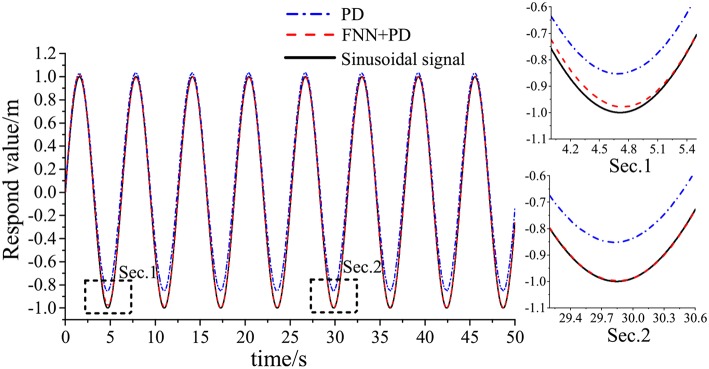
Tracking response of sinusoidal signal.

**Figure 6:**
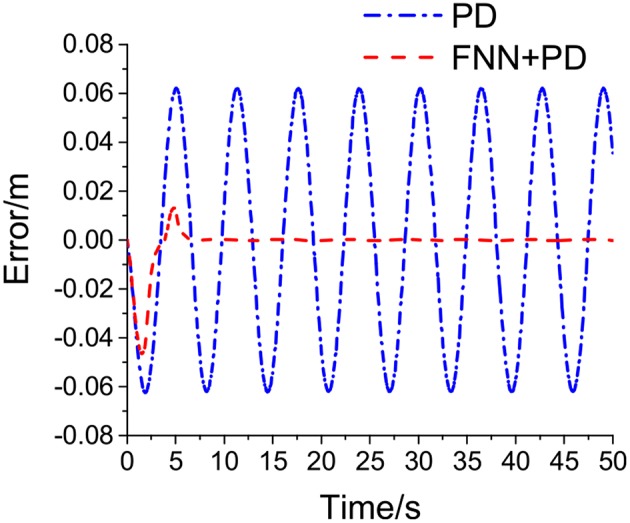
Euclidean error.

**Figure 7:**
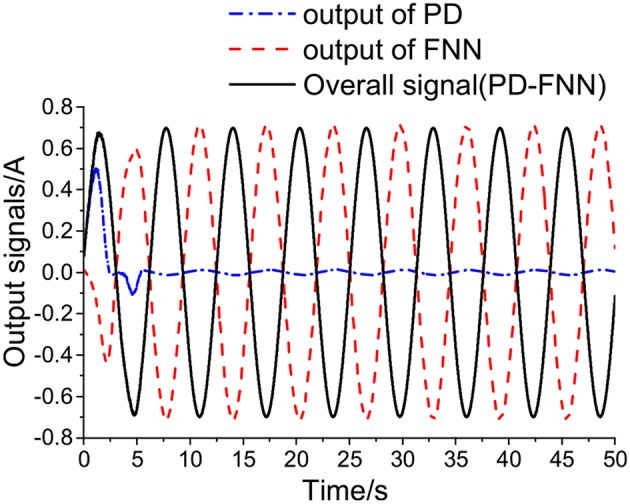
Control signals of PD+FNN.

Table 1 shows the simulation parameters. Figures 4–**7** shows the simulation results without noise. The adaptive learning capabilities of the PD+FNN structure can provide superior performance in different conditions. It is able to solve limitations such as the lack of modeling and existing uncertainties in the environment and is therefore more suitable for real-time applications.

As seen from Figure 4, the PD+FNN controller has a faster and more stable response performance. Figure 5 shows that the controller has a better adaptive learning property to lessen the error gradually. As shown in Figure 6, although the PD controller ensures the error signal is bounded in the neighborhood of zero, significant steady-state errors that occur from internal or external interferences cannot be eliminated. Compared to the PD controller, the PD+FNN controller eliminates the steady-state error.

Figure 7 shows curves of the overall signal (which is defined as *x*_*v*_ = *x*_*c*_−*x*_*f*_), the output of FNN (*x*_*f*_), and the output of the conventional PD controller (*x*_*c*_). As seen in Figure 7, the overall control signal is close to the conventional PD controller at the beginning of the simulation, then the FNN learns the dynamics of the system and takes responsibility for the system. Simultaneously, the output of the PD controller tends to go to zero.

As discussed in the section 2.2, the accumulator energy release process can be regarded as interference according to Gaussian distribution. In order to create different noise levels, four different mean values of the accumulator output flow are chosen, i.e., μ_*v*_ = {100, 150, 200, 300} [L/min]. For the standard deviation, it is selected as σ_*v*_ = 70 L/min.

**Figure 8:**
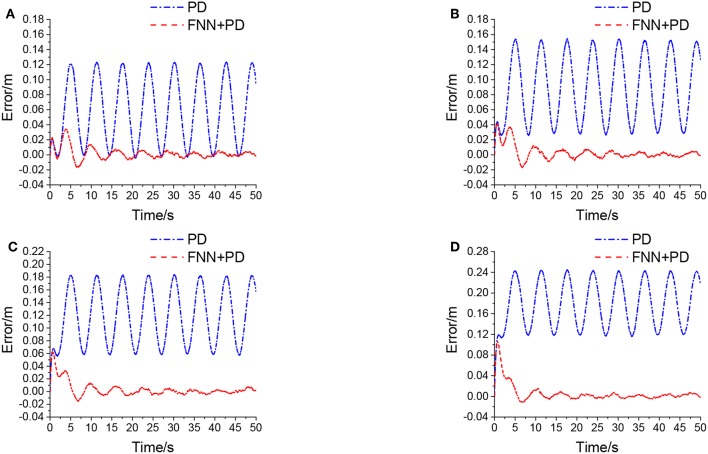
Euclidean error in different levels of noise, **(A)** is μ_*v*_ = 100 *L/min*, σ_*v*_ = 70 *L/min*, **(B)** is μ_*v*_ = 150 *L/min*, σ_*v*_ = 70 *L/min*, **(C)** is μ_*v*_ = 200 *L/min*, σ_*v*_ = 70 *L/min*, **(D)** is μ_*v*_ = 300 *L/min*, σ_*v*_ = 70 *L/min*.

Figure 8 shows the manipulator position errors under PD controller with the noise level from 0.12 to 0.24 m.

Figure 8 shows that the PD controller cannot handle steady-state errors that occur from internal or external interferences. However, the PD+FNN controller can eliminate steady-state errors through an adaptive learning algorithm, and can be used in a real time control to cope with noisy measurements and uncertainties in the system more effectively.

**Figure 9:**
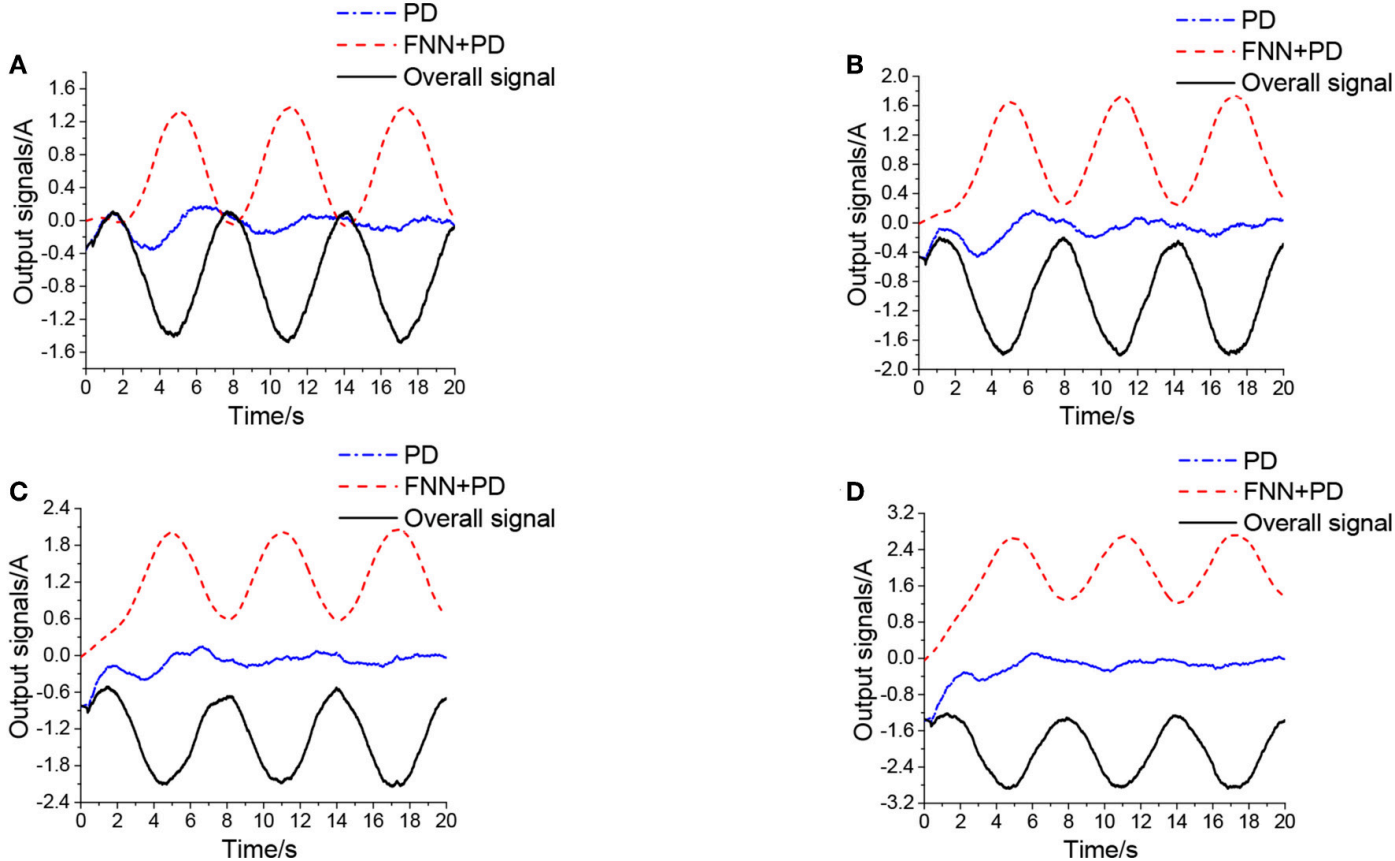
Output signals of PD+ FNN in different levels of noise, **(A)** is μ_*v*_ = 100 *L/min*, σ_*v*_ = 70 *L/min*, **(B)** is μ_*v*_ = 150 *L/min*, σ_*v*_ = 70 *L/min*, **(C)** is μ_*v*_ = 200 *L/min*, σ_*v*_ = 70 *L/min*, **(D)** is μ_*v*_ = 300 *L/min*, σ_*v*_ = 70 *L/min*.

Figure 9 shows the output signals of PD+FNN in different levels of noise. Figure 9, shows that the overall control signal is determined by the FNN controller, and the output signal from PD tends to be close to zero. Therefore, the FNN learns the dynamics of the system and ultimately takes responsibility for the system.

## 5. Experimental Verification

### 5.1. Introduction of Experimental Equipment

**Figure 10:**
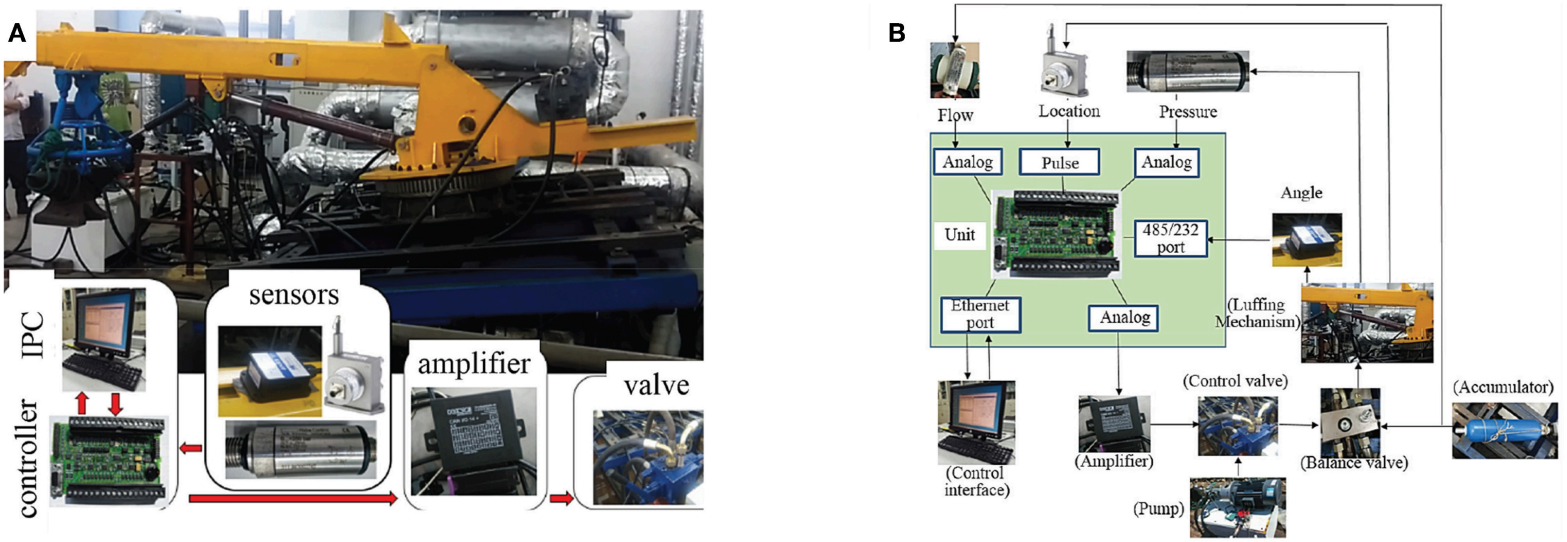
Experimental equipment, **(A)** is the main equipment, **(B)** is the relationships between sensors and the control unit.

The crane experimental model was placed on a swaying table to simulate the sea state. The relationship between each part of manipulators is shown in Figure 10.

The sensor and controller parameters are shown in Table 2.

**Table 2:** Parameters of components.

**Name**	**Content**	**Parameters**
Crane	Weight /*kg*	4.8 × 10^3^
	Maximum system pressure /*Nm*	1.8 × 10^7^
Luffing mechanism	Maximum operating range /*m*	1.5
	Maximum working velocity /*m*·*s*^−*1*^	1
	The pitching angle /°	−20~58
	Maximum lifting weight /*kg*	1.25 × 10^3^
Accumulator	Model type	Bladder
	Volume /**m**^*3*^	6.3 × 10^−3^
	Pre-charge pressure /*Nm*	6 × 10^6^
Cylinder	Bore-rod /*mm*	90–45
Pressure sensor	Range /*Nm*	0−6 × 10^8^
	Response time /*ms*	< 2
	Accuracy	0.3%
	Linearity	≤0.5%
Flow sensor	Range /*m*^*3*^·*h*^−*1*^	0.2–1.2
	Accuracy	±1% Range

### 5.2. Analysis of Step Signal Response

**Figure 11:**
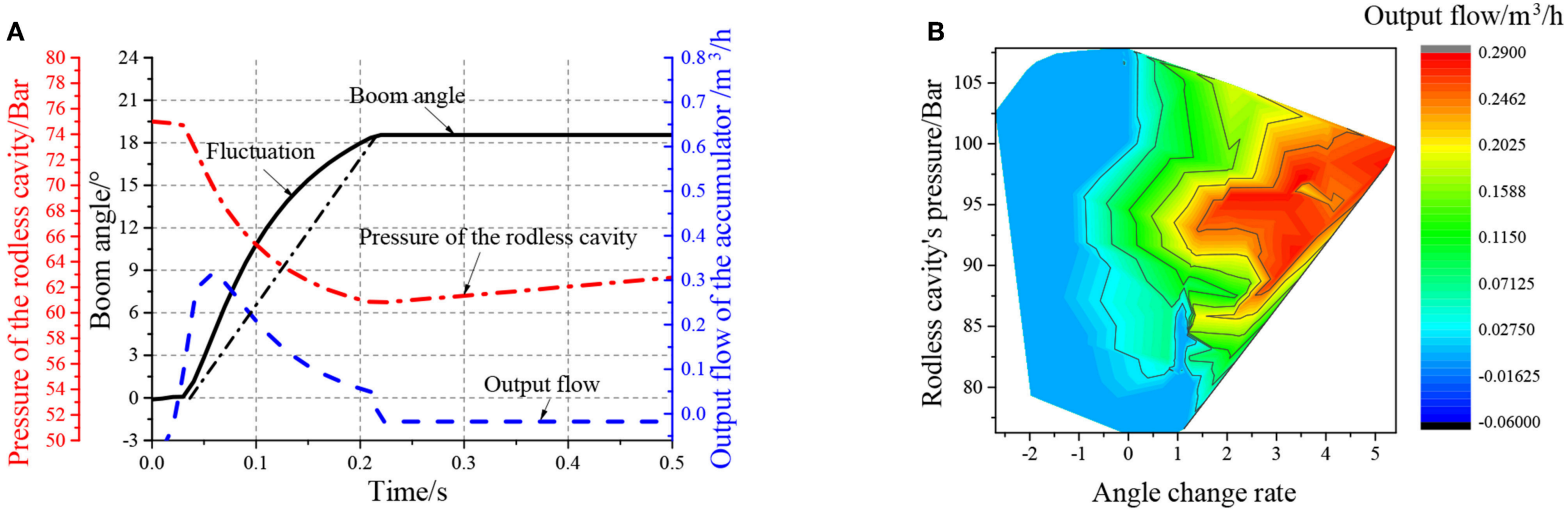
Outstretch simulation of step signal, **(A)** is the fluctuation diagram, **(B)** is the output flow of the cylinder under different angle change rates.

The test results are shown in Figure 11 with the step extension signal.

The release process of the accumulator is shown in Figure 11. When the cylinder is extended, the accumulator releases the oil non-linearly. The cylinder protrudes quickly, and the extension time is about 0.25 s. The color block diagram on the right side of Figure 11 shows that the energy release rate of the accumulator is significantly correlated to the rodless chamber pressure and the cylinder extension speed.

### 5.3. Experimental Strategy and Data Analysis

The swaying table was controlled with a sinusoidal signal in the frequency of 0.11Hz and an amplitude of ±7°. The comparison experiment of automatic wave compensation was performed using a PD algorithm and PD+FNN to control the angular at 0°.

**Figure 12:**
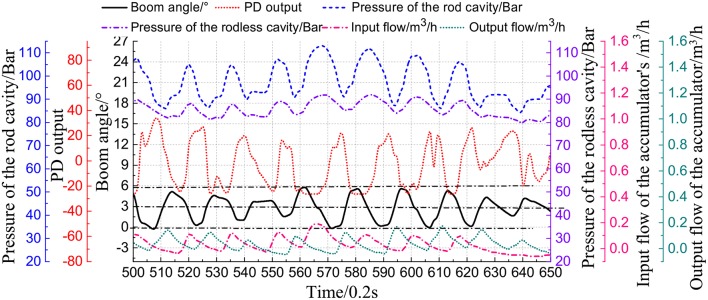
Compensation test with PD strategy.

**Figure 13:**
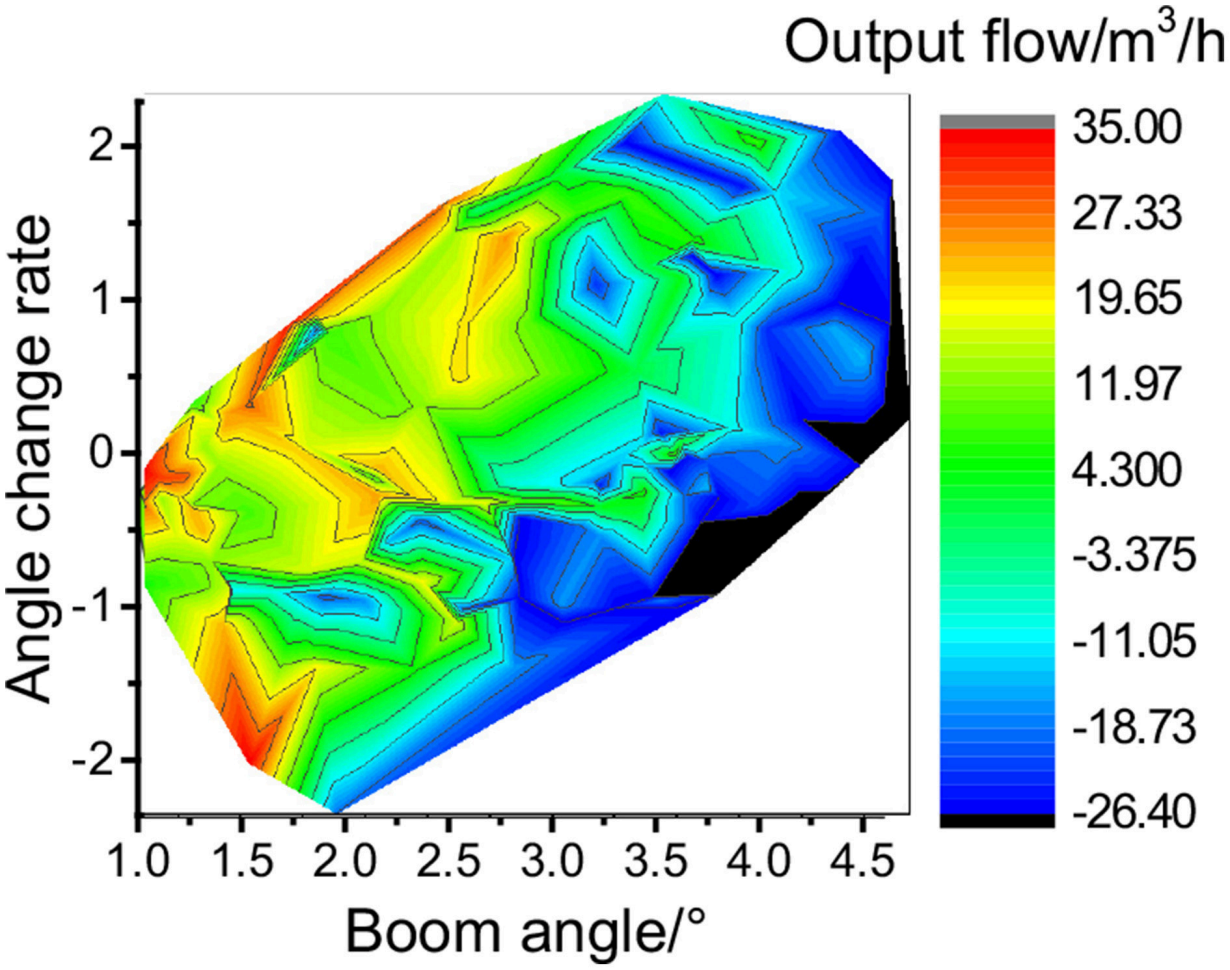
Accumulator output flow with PD strategy.

Figure 12 shows the wave compensation results of the conventional PD controller. During the wave compensation process, the cylinder pressure is stable, and the fluctuation range is approximately 90–105 Bar. However, the expansion and contraction movement of the cylinder is irregular due to the vicious cycle caused by the accumulator intervening. The performance of the cylinder control varies greatly, and the delay is severe. The wave compensation angle error is ±3°. Figure 13 shows that the accumulator flow output is irregular.

**Figure 14:**
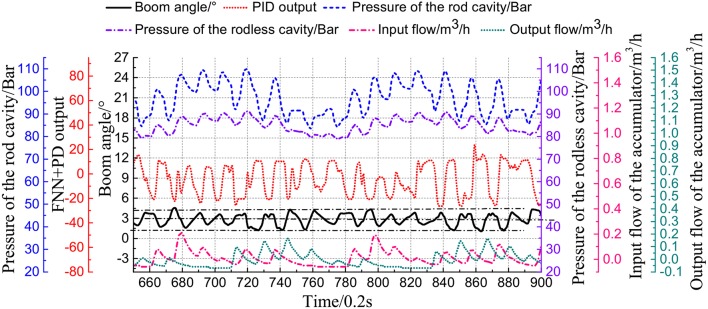
Compensation test with PD+FNN strategy.

Figure 14 shows the experimental results of the PD+FNN controller. The optimization strategy effectively solves the speed shock caused by the accumulator. The cylinder control signal is consistent with the motion of the swinging table, which largely reduces the vibration of the cylinder. The wave compensation effect has been improved, and the angle compensation deviation is stable at ±1°.

**Figure 15:**
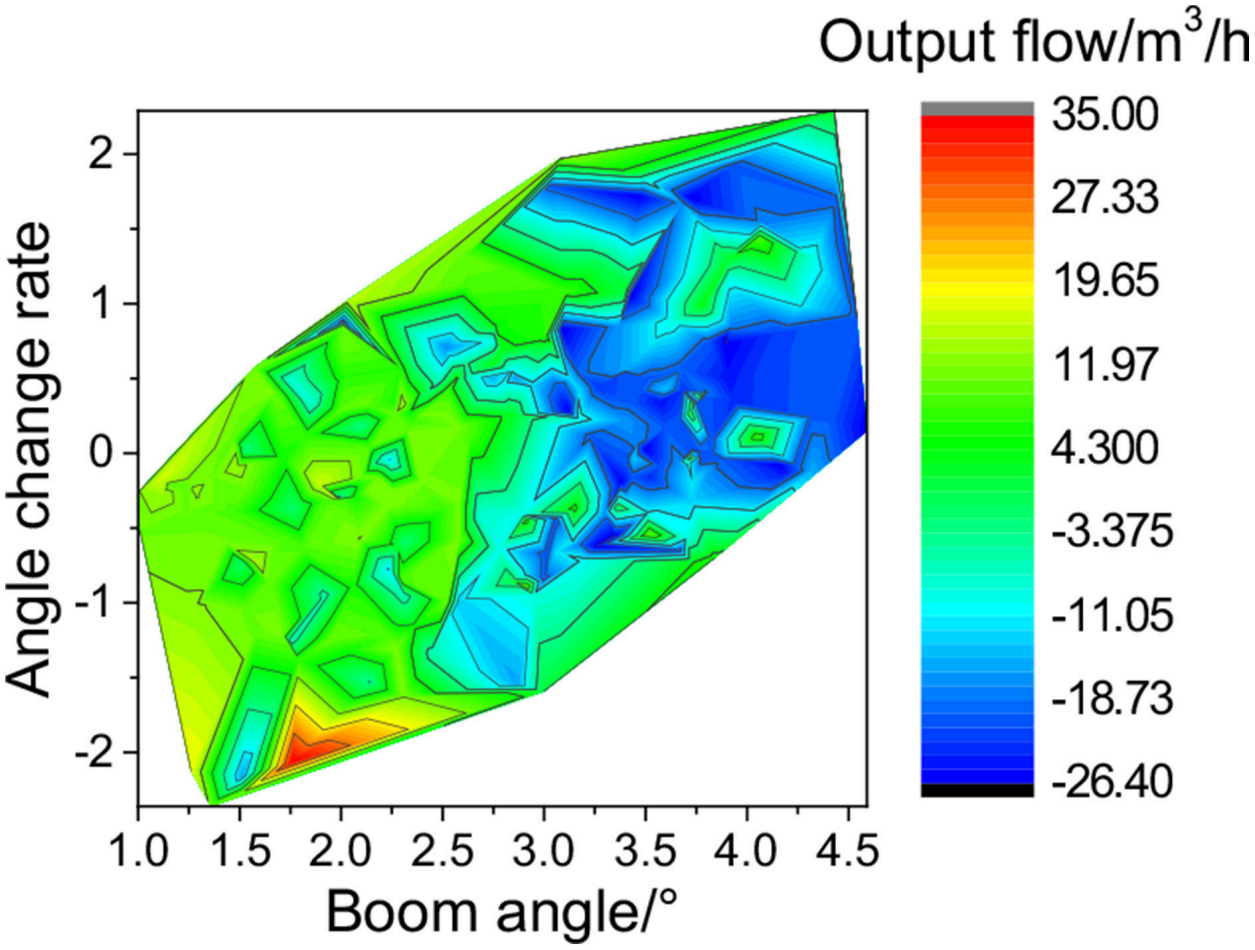
Accumulator output flow with PD+FNN strategy.

Figure 15 shows the highest value of the accumulator flow output concentrated in the negative angle of the cylinder (cylinder extension).

## 6. Conclusion

In this work, a novel control strategy, i.e., PD+FNN approach, is designed to control a shipborne manipulator. Specifically, it is able to handle the high-order non-linearity, time-varying and hysteresis characteristics of the valve-controlled cylinder under the intervention of the accumulator. In addition, it can solve the overshoot generated by the wave compensation process when the accumulator releases energy and the cylinder reacts quickly in the extended stage. Moreover, the presented control strategy is capable of solving the problem of pressure fluctuation. The control precision is improved compared to using a conventional PD controller. Qualitative and quantitative tests on the simulation and real experiments have shown that the proposed controller is capable of significantly reducing steady state-errors and in overcoming the disturbances caused by the accumulator and uncertainties. The deviation angle of compensation is ±1° instead of ±3° compared to the conventional PD controller. We believe that the results of this work will motivate a wider use of the proposed PD+FNN approach, for autonomous collaboration of marine vehicles with a shipborne manipulator.

## Data Availability

All datasets generated for this study are included in the manuscript and/or the supplementary files.

## Author Contributions

ZX and WL conceived and designed the experiments. YW performed the experiments. ZX and YW analyzed the data and wrote the paper.

### Conflict of Interest Statement

The authors declare that the research was conducted in the absence of any commercial or financial relationships that could be construed as a potential conflict of interest.
